# The impact of washed microbiota transplantation on serum gastric function markers: pepsinogen I, pepsinogen II, and Gastrin-17

**DOI:** 10.3389/fnut.2026.1715003

**Published:** 2026-03-12

**Authors:** Youlin Song, Guoqian Liu, Juan Yang, Jiangyan Wang, Shuo Feng, Shenghua Lu, Zhichu Qin, Xingxiang He, Lei Wu

**Affiliations:** 1 Department of Gastroenterology/Microbiota Medicine, Research Center for Engineering Techniques of Microbiota-Targeted Therapies of Guangdong Province, The First Affiliated Hospital of Guangdong Pharmaceutical University, Guangzhou, China; 2 Guangdong Provincial Key Laboratory for Research and Evaluation of Pharmaceutical Preparations, Guangzhou, China

**Keywords:** clinical efficacy, gastrointestinal disorder, gastrointestinal symptoms, gut microbiota, washed microbiota transplantation

## Abstract

**Background and aims:**

Conventional treatment methods for gastric diseases have problems such as drug resistance and recurrence. This study aims to explore whether a new treatment method—Washed Microbiota Transplantation (WMT)—can improve gastric mucosal health.

**Methods:**

The clinical data of patients before and after WMT treatment were collected and analyzed, including serum gastric function markers: gastrin-17 (G-17), pepsinogen I (PGI), pepsinogen II (PGII), and the PGI/PGII ratio (PGR). Inflammatory biomarkers: C-reactive protein (CRP), procalcitonin (PCT), and interleukin-6 (IL-6). Fresh fecal samples were collected at baseline and after WMT treatment and stored at −80 °C until analysis. Gut microbiota profiling was performed using 16S rRNA genes sequencing. Gastrointestinal symptom severity as measured by the Gastrointestinal Symptom Rating Scale (GSRS), and health-related quality of life assessed by the SF-36 physical and mental component summaries (PCS and MCS). The safety and tolerability of WMT were also assessed.

**Results:**

After WMT treatment, serum G-17 and PGI levels decreased significantly within the WMT group (both *p* < 0.05), while PGII demonstrated a downward trend. Notably, in between-group comparisons, only the change in G-17 showed a statistically significant advantage over the control group (*p* = 0.032), whereas differences in PGI, PGII, and PGR were not significant. Inflammatory markers CRP and PCT likewise declined within the WMT group; notably, the magnitude of CRP reduction positively correlated with changes in PGI (*r* > 0.5, *p* < 0.01). Furthermore, 16S rRNA sequencing revealed a significant increase in gut microbial *α*-diversity following WMT, with Chao1, Shannon, and Simpson indices all significantly elevated after the second treatment course compared with baseline (*p* < 0.05); the relative abundances of several key genera were significantly altered. In addition, patients exhibited significant improvement in GSRS scores (*p* < 0.01), and both SF-36 PCS and MCS scores increased markedly compared to baseline (*p* < 0.01). No serious adverse events were observed during the study period; a minority of patients reported mild, transient bloating or diarrhea.

**Conclusion:**

WMT was associated with improvements in gastric mucosal health, gut microbial abundance and diversity, accompanied by reduced inflammation, alleviated gastrointestinal symptoms, improved quality of life, and a favorable safety profile.

## 1 Introduction

Gastrointestinal disorders represent a major global public health concern, encompassing chronic gastritis, peptic ulcer disease, and gastric cancer. These conditions may present with symptoms such as abdominal pain, diarrhea, and hematemesis, and *Helicobacter pylori* infection can drive a progression from chronic inflammation to neoplasia (1, 2). According to the 2022 Global Cancer Statistics report, gastric cancer ranked fifth in incidence and third in mortality among cancers in China (3). In the assessment of gastric mucosal pathology, the triad of serum gastric function biomarkers—gastrin-17 (G-17), pepsinogen I (PGI), and pepsinogen II (PGII)—provides important non-invasive indicators of gastric mucosal status and secretory function (4, 5). Clinically, this panel is primarily applied for risk stratification and for identifying individuals who may warrant further endoscopic evaluation, and it has been used to support the evaluation of conditions such as *H. pylori*–related gastritis, peptic ulcer disease, and gastric neoplasia (6, 7).

The gut microbiota refers to the community of microorganisms residing within the human gastrointestinal tract-including bacteria, archaea, fungi, and viruses—whose equilibrium is essential for maintaining host health (8, 9). In gastric pathology, dysbiosis triggers the release of pro-inflammatory mediators, disrupts the mucosal barrier, and exacerbates mucosal inflammation and immune dysregulation, thereby contributing to the initiation and progression of gastritis, peptic ulceration, and gastric carcinoma through multiple mechanistic pathways (10–12).

Proton pump inhibitors (PPIs) combined with eradication antibiotics remain the standard treatment for gastric diseases; however, issues such as antimicrobial resistance and disease recurrence persist (13, 14). Therefore, there remains an unmet need for novel, effective, and safe therapeutic strategies, such as Washed Microbiota Transplantation (WMT). WMT employs an automated fecal microbiota separation system to process donor stool with the aim of reconstructing the recipient’s gut microbiome, thereby treating gastrointestinal and related disorders while markedly reducing adverse event rates compared to conventional fecal microbiota transplantation (FMT) (15). Several studies have reported potential benefits of WMT in conditions such as inflammatory bowel disease (IBD), metabolic syndrome, and autoimmune disorders (16–20). WMT has demonstrated promising outcomes in alleviating gastric symptoms associated with microbiota dysregulation, including reducing proton pump inhibitor dependence in non-erosive reflux disease (NERD) (21) and serving as an effective intervention for refractory *Helicobacter pylori* infection (22).

The present study aims to evaluate the effects of WMT on serum gastric function indices—G-17, PGI, and PGII—as well as the PGI/PGII ratio (PGR), to investigate the potential for WMT to enhance gastric mucosal health via modulation of the gut microbiota and to provide a scientific rationale for its application in gastric function regulation and the treatment of digestive diseases.

## 2 Materials and methods

### 2.1 Collection of clinical data

During the period from February 2023 to December 2024, electronic medical records were retrospectively collected of patients who received at least two consecutive WMT courses at First Affiliated Hospital of Guangdong Pharmaceutical University, including their basic demographic information (age, sex), serum gastric function biomarkers [G17, PGI, PGII and pepsinogen I/II ratio (PGR)], results from the 13C-urea breath test or serum HP antibody testing, as well as laboratory data such as serum CRP, IL-6, and PCT. Case report form (CRF) data collection was conducted as follows: Trained investigators administered questionnaires in a one-to-one manner during patients’ hospitalization. A dual-checking system was implemented to ensure the integrity of scale entries, followed by the establishment of a dedicated database for electronic data entry. The scales included the GSRS and the SF-36. The SF-36 encompasses eight health domains, which are summarized into two composite scores: the Physical Component Summary (PCS) and the Mental Component Summary (MCS). Given the heterogeneity in primary diagnoses among WMT patients, exploratory subgroup analyses were conducted to evaluate whether disease categories influenced the responsiveness of gastric biomarkers. Additionally, 50 patients treated at our hospital during the same period who did not undergo WMT were enrolled as a non-WMT control group receiving routine clinical care. In this group, demographic variables (age and sex) and serum gastric function biomarkers (G-17, PGI, PGII, and PGR) were collected to serve as a contemporaneous reference for contextual comparison with the WMT cohort. No formal matching was performed for primary diagnosis or baseline biomarker profiles; therefore, between-group analyses were considered exploratory. This study was approved by the Medical Ethics Committee of the First Affiliated Hospital of Guangdong Pharmaceutical University with ethics approval number: 2024 IIT (87).

Definition of data collection time points: Baseline measurements were defined as data obtained prior to the initiation of WMT. Post-treatment data were collected at predefined follow-up time points after each WMT treatment. Specifically, measurements after the first WMT treatment were obtained one month following the first WMT treatment; measurements after the second WMT treatment were obtained one month following the second WMT treatment; and measurements after the third WMT treatment were obtained three months following the third WMT treatment. All post-treatment values were compared with the original baseline measurements obtained before the first WMT treatment. For the non-WMT control group receiving routine clinical care, baseline was defined as the first available serum test and follow-up as the next available test during routine care. Given the non-aligned follow-up schedules, between-group comparisons were restricted to the baseline-to-follow-up change, while multi-course analyses were performed within the WMT cohort.

### 2.2 Inclusion and exclusion criteria

#### 2.2.1 WMT group

Inclusion Criteria: (A) The number of WMT treatment course must be greater than or equal to 2; (B) Age range between 18 and 80 years; (C) Patients who underwent serum gastric function tests.

Exclusion Criteria: (A) History of digestive tract tumors; (B) Severely incomplete medical records.

#### 2.2.2 Control group

Inclusion criteria: (A) Patients with no history of WMT treatment; (B) Aged between 18 and 80 years; (C) Patients who completed serum gastric function tests.

Exclusion criteria: (A) History of gastrointestinal malignancy; (B) Severely incomplete medical records.

### 2.3 Clinical protocol of WMT

Screening of donors: questionnaire screening, physical examination, blood and fecal tests, and other laboratory screenings are conducted on local residents of Guangzhou or school students in the Guangzhou area to exclude samples with mentally disordered, infectious or contagious diseases. Donor stool samples must weigh 50 grams or more and must meet the criteria of categories 3–4 of the Bristol Stool Classification.

Preparation of bacterial liquid: In this study, we used the washing flora transplantation method recommended by the Nanjing Consensus. To prepare the washed microbiota, we prepared a homogenized fecal suspension by adding 500 mL of 0.9% saline to every 100 g of feces in a 1:5 ratio. Next, the fecal suspension was microfiltered using a GenFMTer automated microbiota purification system (FMT Medical, Nanjing, China) to remove particulate matter, parasite eggs, and fungi from it. After microfiltration was completed, the fecal suspension was centrifuged at 1,100 × g for 3 min at room temperature and the centrifuged supernatant was discarded. The microbial pellet was then resuspended in saline and the centrifugation and resuspension process was repeated three times. During the resuspension process, 100 mL of saline was added to 100 g of fecal microbial pellet each time. Finally, the bacterial solution was injected into the patient’s intestines through the digestive tract (120 mL per day for 3 days), depending on the patient’s health status.

Route of WMT: Gastrointestinal injection methods are categorized into two types: upper gastrointestinal tract and lower gastrointestinal tract. There are two upper gastrointestinal methods, including the placement of a nasojejunal TET tube through a gastroscope and the insertion of a Folcke’s spiral nasojejunal tube by hand; and the lower gastrointestinal method involves the placement of an intestinal TET tube through an enteroscope. In this study, the “three–three principle” was used, i.e., each course of treatment consisted of a continuous injection of a suspension of washed flora for 3 days, followed by one course of treatment each month for 3 months (total of three courses of treatment), and then another course of treatment at an interval for 3 months to consolidate the stability of the colonization of the flora, and the whole course of treatment lasted for 5 months.

### 2.4 16S rRNA gene amplicon sequencing

#### 2.4.1 Genomic DNA extraction and quality control

Genomic DNA was extracted and enriched from fecal samples in strict accordance with the manufacturer’s instructions using the E.Z.N.A.® Soil DNA Kit (Omega Bio-tek, USA). To ensure the reliability of downstream analyses, DNA purity and concentration were assessed using a NanoDrop 2000 spectrophotometer (Thermo Fisher Scientific, USA). Based on these measurements, quality control criteria for DNA samples were established to meet the technical requirements for subsequent analyses.

#### 2.4.2 16S rRNA gene sequencing workflow

The V3–V4 hypervariable regions of the bacterial 16S rRNA gene were selected as the target regions and amplified using the specific primer pair 338F (5′-ACTCCTACGGGAGGCAGCAG-3′) and 806R (5′-GGACTACHVGGGTWTCTAAT-3′). PCR amplification was performed in a 20 μL reaction volume containing 4 μL of 5 × TransStart FastPfu buffer, 2 μL of 2.5 mM dNTPs, 0.8 μM of each forward and reverse primer, 0.4 U of FastPfu DNA polymerase, and 10 ng of template DNA. The thermal cycling conditions were as follows: initial denaturation at 95 °C for 30 s; followed by 27 cycles of denaturation at 95 °C for 30 s, annealing at 55 °C for 30 s, and extension at 72 °C for 45 s; with a final extension at 72 °C for 5 min. PCR products were verified by 1.5% agarose gel electrophoresis to confirm the expected amplicon size and were subsequently subjected to paired-end sequencing on the Illumina MiSeq PE300 platform by Novogene Bioinformatics Technology Co., Ltd.

#### 2.4.3 Amplicon sequence processing and bioinformatics analysis

Raw sequencing reads were merged using FLASH (v1.2.11) and quality-filtered using FASTP (v0.19.6). Sequence processing was conducted using the QIIME2 pipeline (version 2020.2). Denoising and chimera removal were performed using the DADA2 algorithm, yielding amplicon sequence variants (ASVs). To ensure comparability among samples, all sequences were rarefied to 4,000 reads per sample. Taxonomic annotation was performed using the SILVA 16S rRNA database (version 138), and microbial community structure analyses were completed using the Novomagic cloud platform.

### 2.5 Statistical analysis

Data were processed using GraphPad Prism 10.0 and SPSS 29.0. Continuous variables were first examined for normality. Normally distributed data are presented as mean ± standard deviation, whereas non-normally distributed data are expressed as median (interquartile range). For within-subject comparisons between baseline and post-treatment values, a paired *t*-test was used for normally distributed data, and the Wilcoxon signed-rank test was applied for non-normally distributed data. Between-group comparisons were performed using the independent-samples *t*-test or the Mann–Whitney *U* test, as appropriate. Categorical variables are reported as counts and percentages and were compared using the χ^2^ test or Fisher’s exact test, as appropriate. Correlation analyses were conducted using Pearson or Spearman correlation coefficients depending on data distribution. All statistical tests were two-sided, and *p* values < 0.05 were considered statistically significant. In figures and tables, *p* < 0.05 is indicated by *, *p* < 0.01 by **, and *p* > 0.05 by ns.

## 3 Results

### 3.1 Patient demographics

Based on the inclusion and exclusion criteria, a total of 2 or more WMT patients 73 who underwent WMT from February 2023 to December 2024 at the First Hospital of Guangdong Pharmaceutical University were included. Another 50 patients who did not undergo WMT under conventional treatment in our department from January 2023 to December 2024 were included and set as the control group. As shown in Table 1, the median age of patients in the WMT group was 49.00 (37.50, 62.00) years, and 55.50 (49.00, 61.25) years in the control group, and the difference between the groups was not statistically significant (*p* = 0.055). In terms of gender distribution, 46.58% of the WMT group was male and 50.00% of the control group was male, with similar gender composition in both groups (*p* = 0.709).

**Table 1:** Comparison of baseline characteristics between the two groups of patients.

Basic information	WMT group (*n* = 73)	Control group (*n* = 50)	*p*-value
Age (years)	49.00 (37.50, 62.00)	55.50 (49.00, 61.25)	0.055
Sex (M, %)	34 (46.58%)	25 (50.00%)	0.709

### 3.2 Primary diagnosis of patients

Among 73 cases of patients who underwent WMT, the most common indication was functional bowel disease. Among 47 patients (64.38%) opted for WMT because of functional bowel disease. Followed by organic gastrointestinal disorders 8 (10.96%), endocrine metabolic diseases cases (5.5%), tumor related diseases 6 (8.22%), neurological and psychiatric diseases 5 (6.85%), and autoimmune disorders 3 (4.11%) as shown in Table 2. Among the 50 patients in the control group, 26 cases (52.00%) underwent routine treatment at our hospital due to functional bowel disorders. This was followed by 16 cases (32.00%) of organic gastrointestinal diseases and 8 cases (16.00%) of other conditions, as detailed in Table 3.

**Table 2:** Distribution of primary diagnoses of WMT patients.

Disease category	Illnesses	Number of examples	Percentage (%)
Functional bowel disease	Irritable bowel syndrome (IBS)	25	32.25%
	Functional dyspepsia	9	12.33%
	Functional diarrhea	4	5.48%
	Functional constipation	9	12.33%
Organic gastrointestinal diseases	Infectious enteritis	2	2.73%
	Inflammatory bowel disease	5	6.85%
	Reflux esophagitis	1	1.37%
Endocrine metabolic diseases	Type 2 diabetes with poor glycemic control	2	2.73%
	Obesity	1	1.37%
	Gout	1	1.37%
Tumor-related diseases	Sequelae of radiotherapy for malignant tumors	6	8.22%
Neurological and mental diseases	Amyotrophic lateral sclerosis (ALS)	3	4.11%
	Bipolar disorder	1	1.37%
	Idiopathic tremor	1	1.37%
Autoimmune disease	Psoriasis	2	2.73%
	Autoimmune encephalitis	1	1.37%
(Grand) total		73	100%

**Table 3:** Distribution of primary diagnoses in the control group.

Disease category	Illnesses	Number of examples	Percentage (%)
Functional bowel disease	Irritable bowel syndrome (IBS)	8	16.00%
	Functional dyspepsia	12	24.00%
	Functional diarrhea	2	4.00%
	Functional constipation	4	8.00%
Organic gastrointestinal diseases	Gastric ulcer	4	8.00%
	Esophageal varices	1	2.00%
	Gastric polyp	1	2.00%
	Colon polyp	7	14.00%
	Reflux esophagitis	2	4.00%
	Hemorrhagic internal hemorrhoids	1	2.00%
Other	Pancreatitis	6	12.00%
	Weight loss	2	4.00%
(Grand) total		50	100%

### 3.3 Effect of WMT on gastric serum function in patients

**Figure 1:**
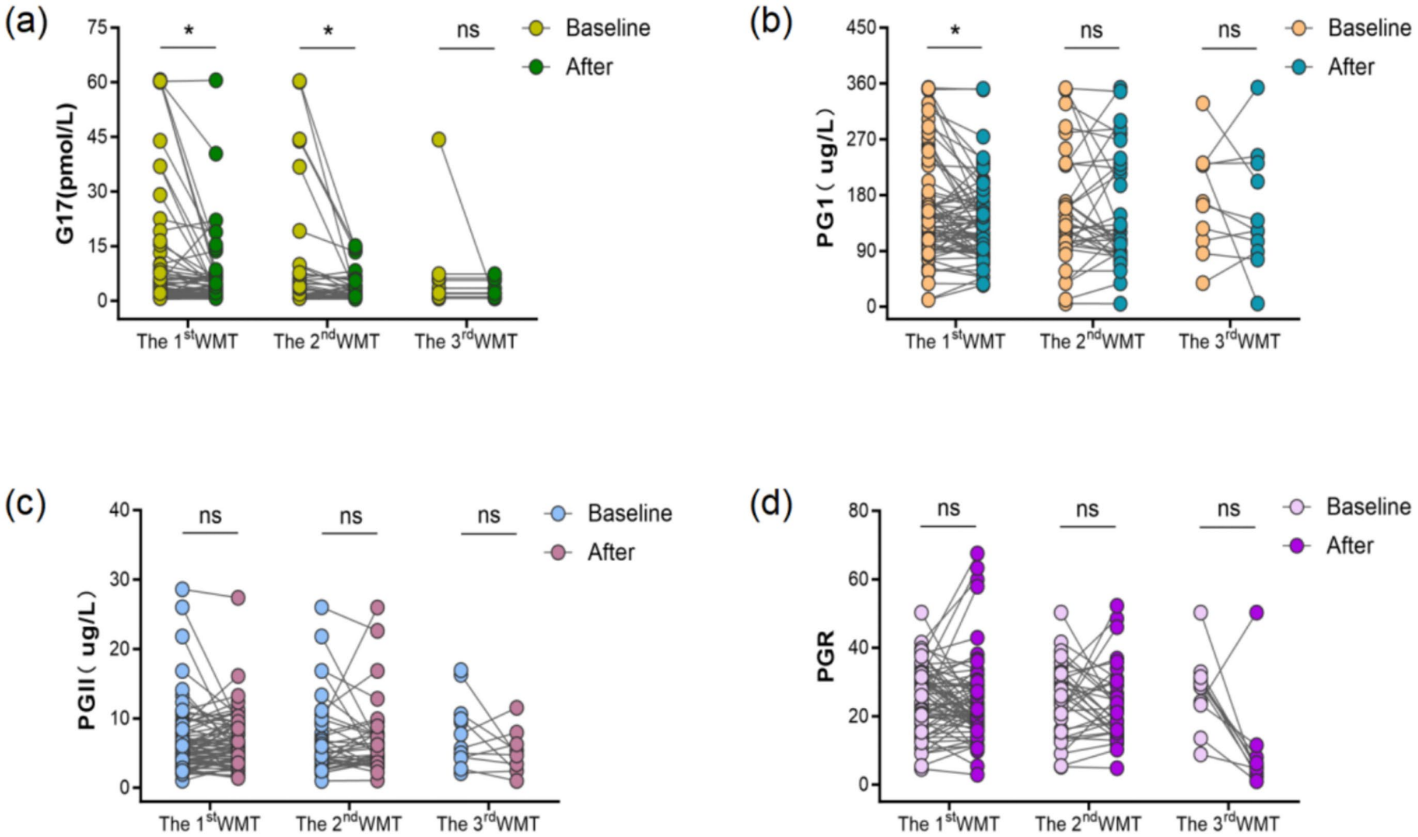
Effect of WMT on patient G-17, PGI, PGII, and PGR. Data after each WMT session were paired with baseline data for comparison. **(a)** Differences in gastrin G17 expression after different courses; **(b)** Comparison of pepsinogen I after different courses; **(c)** Comparison of pepsinogen I after different courses; **(d)** Comparison of the ratio of pepsinogen I to PG II after different courses. *p < 0.05; ns: no significant difference.

Differential analysis of gastric serum function in patients before and after WMT. Changes in the levels of the three gastric function items (G-17, PGI, PGII and PGR) at baseline and after different WMT sessions are shown in Table 4. The distribution of strong responders across disease categories for each gastric function biomarker is shown in Table 5. Strong responders were predominantly observed in patients with functional bowel disease for ΔG-17, ΔPGI, ΔPGII, and ΔPGR. However, no significant differences in the distribution of strong and non-strong responders across disease categories were detected for ΔG-17 (*p* = 0.587), ΔPGI (*p* = 0.945), or ΔPGII (*p* = 0.945). For ΔPGR, a borderline association was observed but did not reach statistical significance (*p* = 0.057). G-17 levels decreased significantly after the first WMT and second WMT treatments (both *p* < 0.05), whereas no statistically significant difference was observed after the third WMT treatment (*p* > 0.05). PGI levels decreased significantly after the first WMT treatment (*p* < 0.05), with no significant changes observed after subsequent treatments (*p* > 0.05). No significant differences were observed in PGII or PGR levels before and after WMT (*p* > 0.05) (Figure 1).

**Table 4:** Levels of serum gastric function changes in WMT patients.

Items	Base line (in geodetic survey) (*n* = 73)	WMT 1 course (*n* = 73)	WMT 2 course (*n* = 35)	WMT 3 course (*n* = 11)
G17 (pmol/L)	3.38 (1.27, 8.04)	2.69 (1.43, 6.51)	2.39 (1.14, 4.35)	1.89 (1.15, 4.25)
PGI (μg/L)	137.50 (94.69, 183.60)	133.00 (88.54, 167.10)	120.50 (88.44, 230.5)	122.90 (89.22, 231.50)
PGII (μg/L)	5.64 (4.02, 8.54)	5.90 (4.10, 8.99)	5.09 (3.70, 7.60)	4.75 (2.43, 7.97)
PGR	26.32 (18.19, 31.63)	20.93 (17.10, 29.02)	21.62 (15.33, 30.32)	29.75 (15.23, 37.63)

**Table 5:** Distribution of strong responders across disease categories for gastric function biomarkers after WMT (*n* = 73).

Items	Disease category	Strong responders, *n* (%)	Non-strong responders, *n* (%)	*p* value
ΔG17	Functional bowel disease	10 (55.5%)	37 (67.27%)	0.587
	Organic gastrointestinal diseases	4 (22.2%)	4 (7.27%)	
	Endocrine metabolic diseases	1 (5.55%)	3 (5.45%)	
	Tumor-related diseases	1 (5.55%)	5 (9.09%)	
	Neurological and mental diseases	1 (5.55%)	4 (7.27%)	
	Autoimmune disease	1 (5.55%)	2 (3.64%)	
ΔPGI	Functional bowel disease	11 (61.11%)	36 (65.45%)	0.945
	Organic gastrointestinal diseases	3 (16.67%)	5 (9.09%)	
	Endocrine metabolic diseases	1 (5.55%)	3 (5.45%)	
	Tumor-related diseases	1 (5.55%)	5 (9.09%)	
	Neurological and mental diseases	1 (5.55%)	4 (7.27%)	
	Autoimmune disease	1 (5.55%)	2 (3.64%)	
ΔPGII	Functional bowel disease	12 (66.67%)	35 (63.64%)	0.945
	Organic gastrointestinal diseases	2 (11.11%)	6 (10.90%)	
	Endocrine metabolic diseases	1 (5.55%)	3 (5.45%)	
	Tumor-related diseases	1 (5.55%)	5 (9.09%)	
	Neurological and mental diseases	2 (11.11%)	3 (5.45%)	
	Autoimmune disease	0	3 (5.45%)	
ΔPGR	Functional bowel disease	11 (61.11%)	36 (65.45%)	0.057
	Organic gastrointestinal diseases	1 (5.55%)	7 (12.73%)	
	Endocrine metabolic diseases	1 (5.55%)	3 (5.45%)	
	Tumor-related diseases	0	6 (10.91%)	
	Neurological and mental diseases	4 (22.22%)	1 (1.82%)	
	Autoimmune disease	1 (5.55%)	3 (5.45%)	

Values are presented as *n* (%), with percentages calculated within each response category. Strong responders were defined separately for each biomarker as patients whose decrease in the corresponding parameter (Δ value) ranked within the highest 25% of the change distribution following WMT. *p* values were calculated using Fisher’s exact test.

Comparison of serum gastric function three items and PGR difference before and after treatment between control group and WMT group. Comparison of the differences in serum gastric function indexes (G17, PGI, PGII and PGR) between the two groups of patients before and after treatment demonstrated that: the median differences (interquartile spacing) in G17, PGI, PGII, and PGR in the WMT group were −0.49 (−2.68, 1.00) pmol/L, −4.59 (−44.48, 19.11) μg/L, −0.09 (−1.19, 1.81) μg/L and 0.13 (−8.37, 5.17), while the corresponding index differences in the control group were 0.22 (−2.60, 9.13) pmol/L, −3.42 (−44.45, 28.92) μg/L, 0.26 (−2.30, 2.18) μg/L, and −1.09 (−9.23, 6.69), respectively. Analyzed by Mann–Whitney *U* test, the absolute value of G17 difference in the WMT group was significantly higher than that in the control group (*p* < 0.05), and there was no significant difference in the difference of PGI, PGII and PGR between the two groups (*p* > 0.05) in Table 6.

**Table 6:** Comparison of differential gastric function between the two groups of patients.

Items	WMT group (*n* = 73)	Control group (*n* = 50)	*p* value
ΔG17 (pmol/L)	−0.49 (−2.68, 1.00)	0.22 (−2.60, 9.13)	0.032
ΔPGI (μg/L)	−4.59 (−44.48, 19.11)	−3.42 (−44.45, 28.92)	0.812
ΔPGII (μg/L)	−0.09 (−1.19, 1.81)	0.26 (−2.30, 2.18)	0.805
ΔPGR	0.13 (−8.37, 5.17)	−1.09 (−9.23, 6.69)	0.954

### 3.4 Effects of WMT on the patients gut microbiota

**Figure 2:**
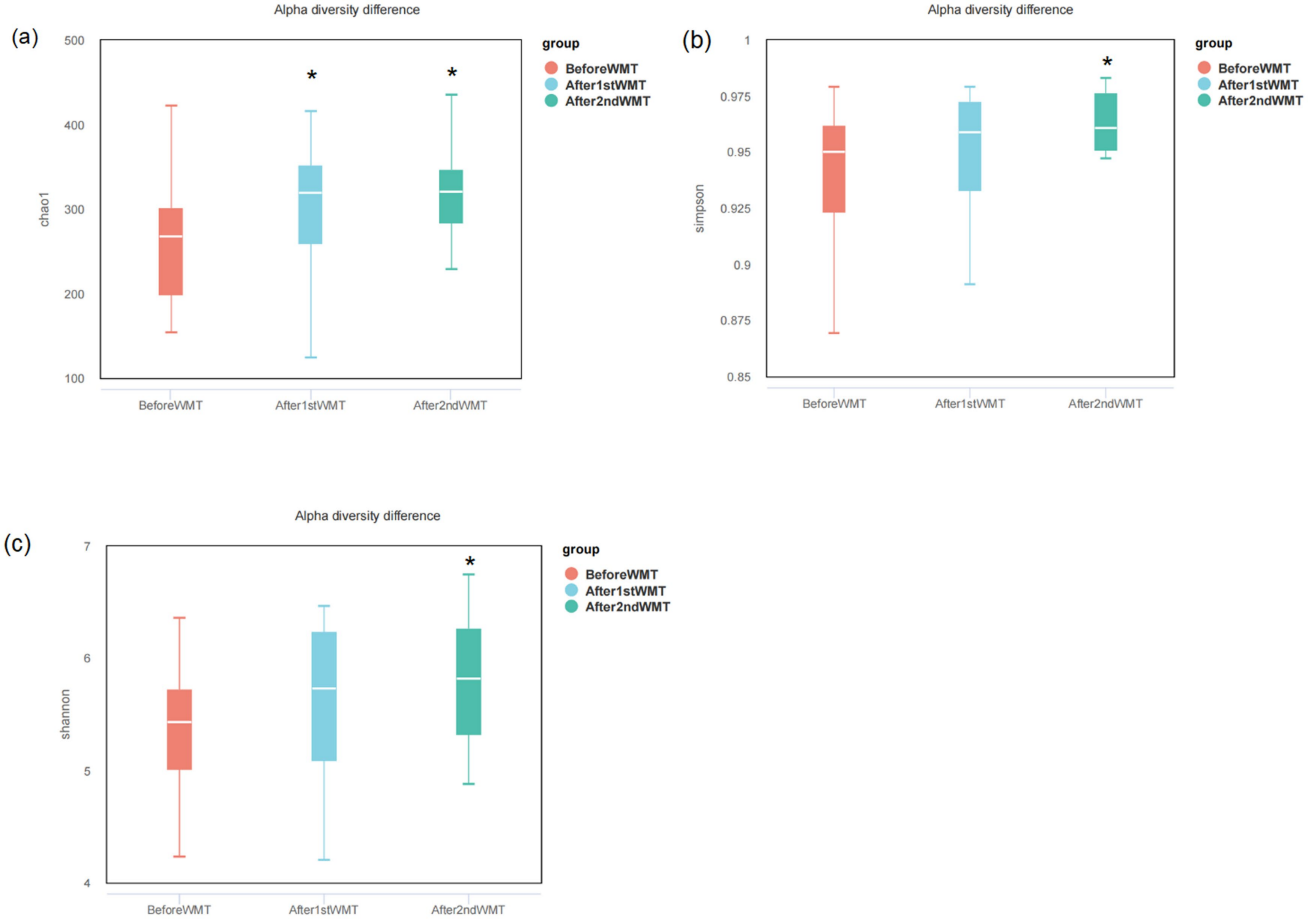
Alpha diversity indices of the fecal microbiota at the genus level before and after WMT. **(a)** Chao1 index; **(b)** Simpson index; **(c)** Shannon index. *p < 0.05 compared with baseline.

**Figure 3:**
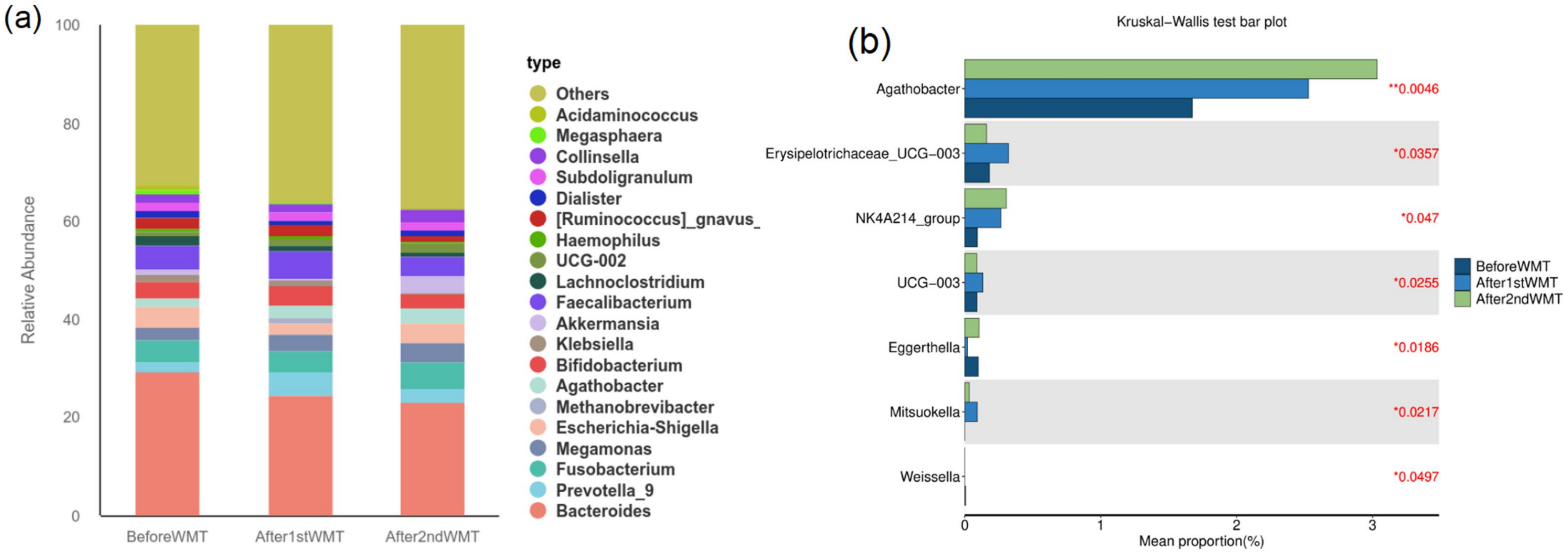
Composition and differential analysis of the fecal microbiota before and after WMT. **(a)** Bar plot of relative abundance at the genus level; **(b)** intergroup differences analyzed using the Kruskal–Wallis test.

**Figure 4:**
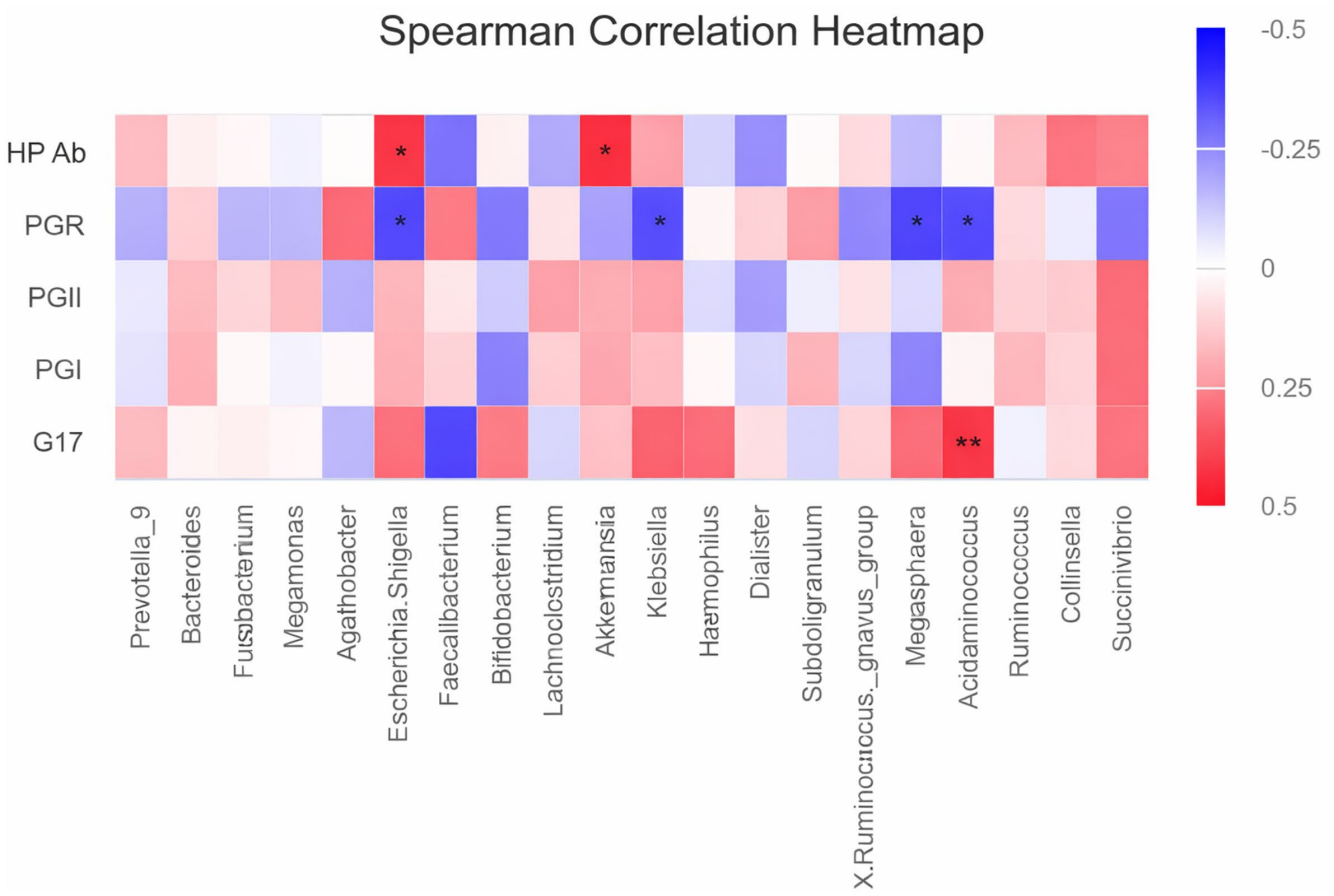
Heatmap of Spearman correlations between baseline serum gastric function parameters (G-17, PGI, PGII), PGR, HP antibodies, and the fecal microbiota at the genus level.

Fecal samples from 34 patients before and after WMT were analyzed using 16S rRNA genes sequencing. As shown in Figure 2, changes in alpha diversity indices at the genus level were evaluated at baseline, after the first WMT treatment, and after the second WMT treatment. Compared with baseline, the Chao1 index was significantly increased after the first WMT treatment (*p* < 0.05). After the second WMT treatment, the Chao1, Simpson, and Shannon indices were all significantly higher than those at baseline (*p* < 0.05), indicating an overall increase in microbial richness and diversity following WMT. The relative abundance of the major bacterial genera in fecal samples before and after WMT is shown in Figure 3a. The results of intergroup difference analysis using the Kruskal–Wallis test are presented in Figure 3b, which demonstrated that the relative abundances of *Agathobacter*, *Erysipelotrichaceae*_UCG-003, NK4A214_group, UCG-003, *Eggerthella*, *Mitsuokella*, and *Weissella* were significantly altered after WMT (*p* < 0.05). Figure 4 illustrates the Spearman correlation analysis between baseline serum gastric function parameters (G-17, PGI, and PGII), PGR, HP antibodies, and the fecal microbiota at the genus level. The results demonstrated that HP antibody levels were positively correlated with *Escherichia–Shigella* and *Akkermansia* (*p* < 0.05). PGR was negatively correlated with *Escherichia–Shigella*, *Klebsiella*, *Megasphaera*, and *Acidaminococcus* (*p* < 0.05). In addition, G-17 was significantly positively correlated with *Acidaminococcus* (*p* < 0.01).

### 3.5 Effect of WMT on inflammatory markers in patients and its correlation with changes in gastric function

**Figure 5:**
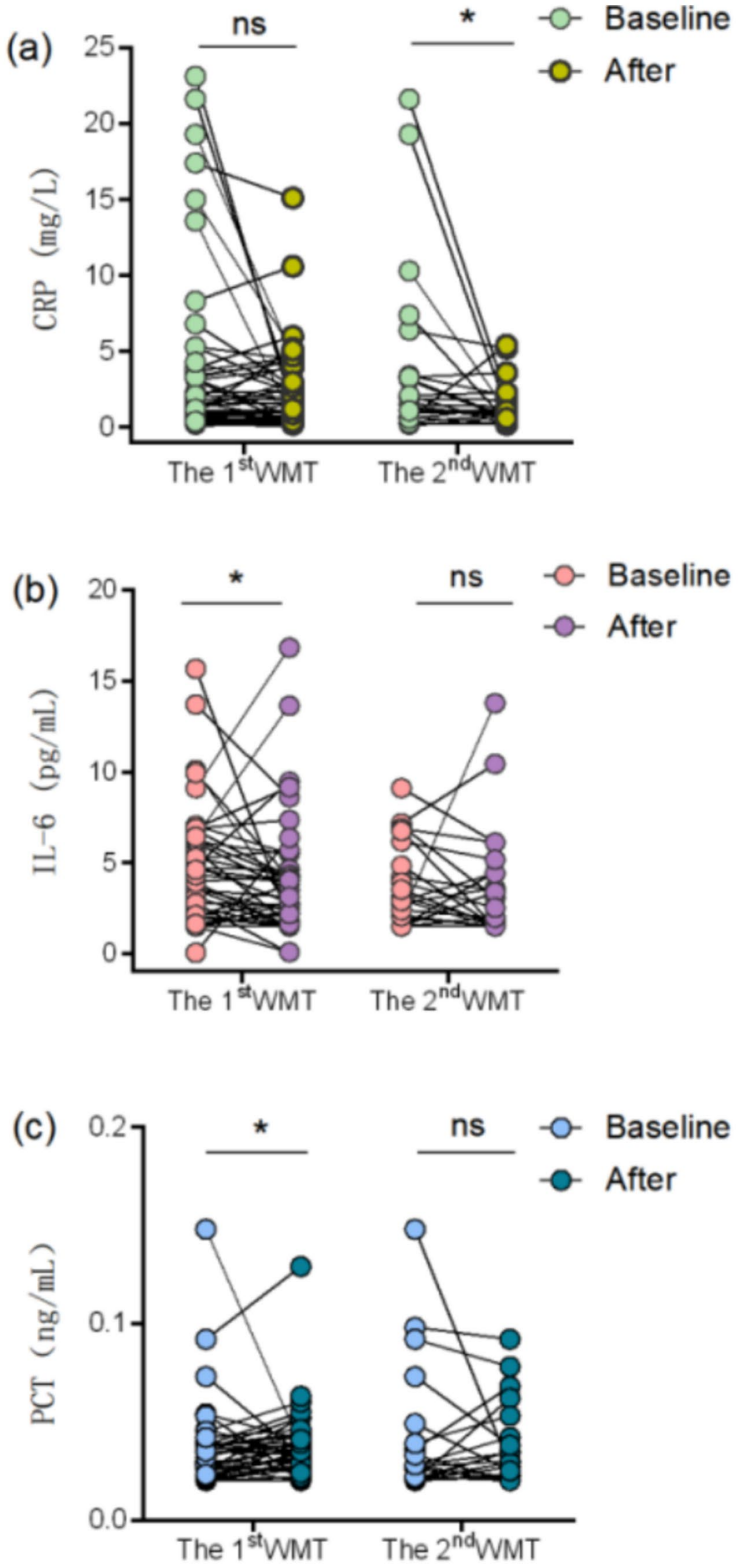
Effect of WMT on Patient C RP, IL-6 and PCT. Pairwise comparisons of C RP, IL-6 and PCT data after each WMT session with baseline data. **(a)** Change in C RP after washout colony transplantation; **(b)** change in IL-6 after washout colony transplantation; **(c)** change in PCT after washout colony transplantation. *p < 0.05; ns not significantly different.

A total of 73 patients were included in the WMT cohort. Among them, inflammatory biomarker data were available for 48 patients after the and for 24 patients after the second WMT treatment. At each treatment course, the numbers of patients included in the analyses were identical for all three inflammatory biomarkers. Serum levels of C-reactive protein (CRP), interleukin-6 (IL-6), and procalcitonin (PCT) were compared with baseline values in patients with available data. As shown in Figure 5, serum CRP decreased significantly after the second WMT treatment (*p* < 0.05). IL-6 and PCT levels both decreased significantly after the first WMT treatment (both *p* < 0.05).

Correlation between changes in inflammatory markers and serum gastric function markers in patients from baseline to after the first WMT treatment. After Pearson’s correlation analysis, the correlations between the indicators and their statistical significance were as follows: CRP demonstrated a significant positive correlation with PGI (*r* = 0.46, *p* = 0.001), suggesting that there may be some correlation between the two. The correlation between CRP and G-17 (*r* = 0.23, *p* = 0.136), PGII (*r* = 0.20, *p* = 0.190), and PGR (*r* = 0.10, *p* = 0.516) correlations did not reach statistical significance. Further analysis revealed that IL-6 was not significantly correlated with all variables (*p* > 0.05), with a weak positive correlation with PGII as (*r* = 0.22, *p* = 0.139). In addition, the correlation coefficients of PCT with G-17, PGI, PGII, and PGR were all close to zero (|*r*| ≤ 0.16) and the *p*-values were all greater than 0.05, indicating that PCT was not significantly associated with these variables in Table 7.

**Table 7:** Pearson correlation coefficients (*r*) and significance levels (*p*) between indicators (*n* = 48).

Items	G-17	PGI	PGII	PGR
CRP	0.23 (*p* = 0.136)	0.46 (*p* = 0.001)	0.20 (*p* = 0.190)	0.10 (*p* = 0.516)
IL-6	0.20 (*p* = 0.188)	0.17 (*p* = 0.258)	0.22 (*p* = 0.139)	−0.09 (*p* = 0.561)
PCT	−0.05 (*p* = 0.757)	0.16 (*p* = 0.283)	0.09 (*p* = 0.550)	−0.02 (*p* = 0.897)

### 3.6 Effect of WMT on patients’ gastrointestinal symptoms (GSRS score)

**Figure 6:**
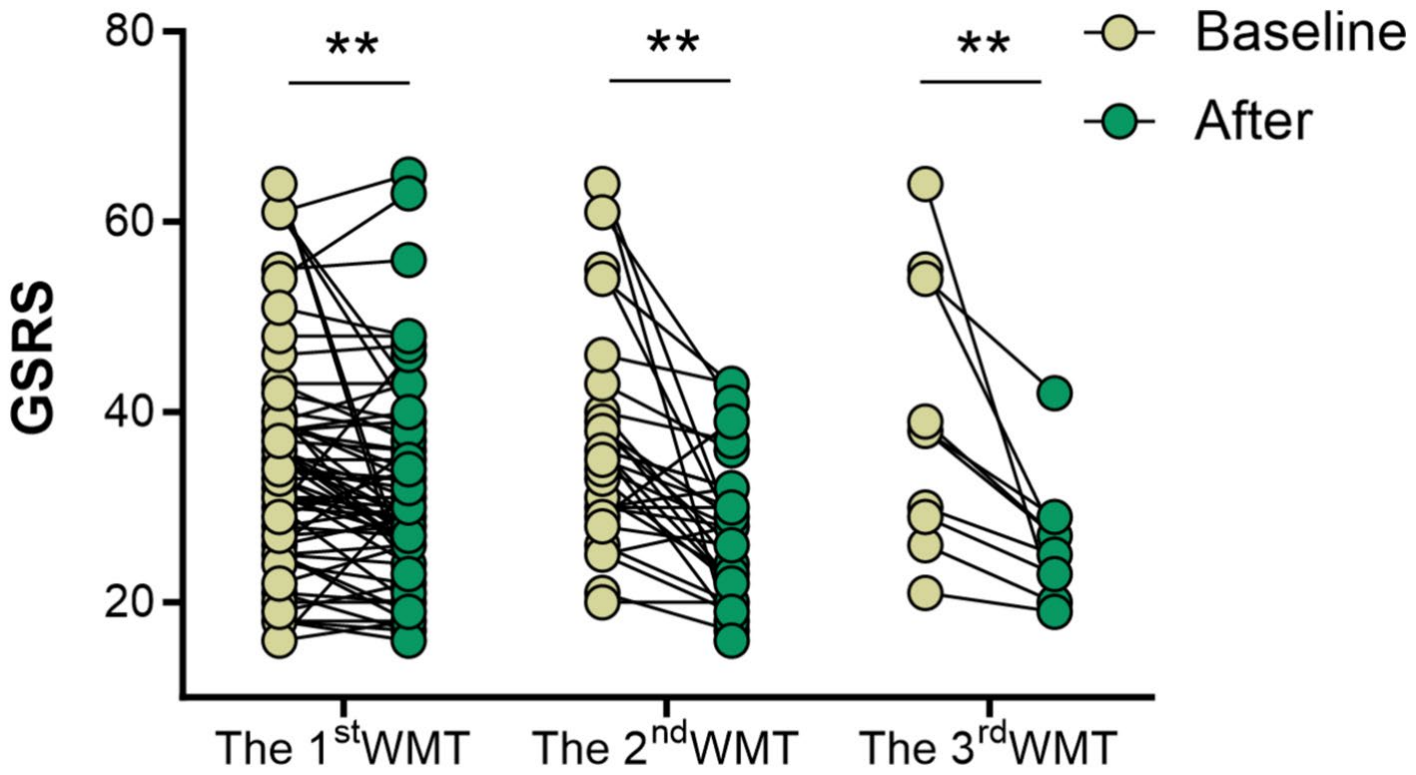
Effect of WMT on patients’ gastrointestinal symptoms (GSRS scores). First WMT treatment (*n* = 64), the second WMT treatment (*n* = 30), the third WMT treatment (*n* = 10).

As shown in Figure 6, GSRS scores decreased from 33.50 (26.00, 39.00) to 29.00 (24.00, 36.75) after the 1st WMT, from 36.00 (29.75, 40.75) to 28.00 (22.00, 32.00) after the 2nd WMT, and from 38.00 (28.25, 54.25) after the third WMT treatment to 26.00 (20.00, 27.50), all differences were statistically significant (*p* < 0.01).

### 3.7 The effect of WMT on patients’ SF-36 quality of life scale physical health total score and mental health total score

**Figure 7:**
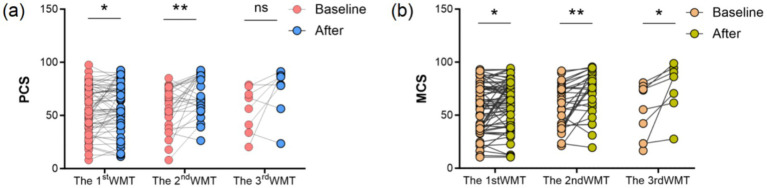
Effect of WMT on patients’ SF-36 Quality of Life Scale Physical Health (PCS) **(a)** and Mental Health (MCS) **(b)**. The first WMT treatment (*n* = 65), the second WMT treatment (*n* = 29), the third WMT treatment (*n* = 8).

In this study, the SF-36 Quality of Life Scale Physical Health Total Score (PCS) and Mental Health Total Score (MCS) were assessed in patients with WMT. PCS was significantly elevated from baseline after the first WMT treatment (*p* < 0.05), further improved after 2 courses of WMT (*p* < 0.01), and did not show any significant change after 3 courses of WMT (*p* > 0.05). As shown in Figure 7, The MCS demonstrated significant increases from baseline following the first, second, and third WMT treatments (*p* < 0.05 or *p* < 0.01).

### 3.8 Adverse reaction

In this study, 73 cases of WMT patients completed a cumulative total of 187 courses of WMT treatment. The incidence of adverse reactions was 4.81%, including diarrhea in 3 cases, dizziness in 3 cases, headache in 1 case, fever in 1 case, and vulvar itching in 1 case, and no other serious adverse reactions occurred.

## 4 Discussion

In this study, we systematically investigated changes in serum gastric function, gut microbiota composition, inflammatory markers, gastrointestinal symptoms, and quality of life following WMT treatment. The findings indicate that WMT was associated with increased gut microbial abundance and diversity, accompanied by significant reductions in serum G-17 and PGI levels as well as inflammatory markers. Moreover, changes in the inflammatory marker C-reactive protein (CRP) were positively correlated with changes in PGI. Clinically, gastrointestinal symptom scores were significantly alleviated, SF-36 quality-of-life scores were increased, and WMT exhibited a favorable safety profile.

Serum G-17 and PGI are important biomarkers for gastric cancer screening and have demonstrated high sensitivity and specificity, particularly in the detection of early and advanced gastric cancer (6, 23). G17 (gastrin-17) is a gastrointestinal peptide secreted by gastric sinus G-cells, which regulates gastric acid secretion mainly through a negative feedback mechanism. The secretion of G17 is inhibited when gastric acid is excessive, and the level of G17 may be increased in low acid state or delayed gastric emptying (24, 25). PGI is secreted by the main cells of the gastric body, and its serum level reflects the structural and functional status of the gastric body mucosa. Decreased levels of PGI are suggestive of gastric glandular atrophy, which is commonly seen in atrophic gastritis and related precancerous lesions, while elevated levels can be seen in increased acid secretion or inflammatory diseases of the gastric mucosa (26). HP infection is one of the main causes of gastric mucosal lesions, and elevated PGI levels are usually associated with poor HP clearance (6) As an indirect indicator reflecting gastric mucosal function and gastric acid status, G17 combined with pepsinogen assay can be used for risk assessment of gastric mucosal lesions, especially in the screening of atrophic gastritis and gastric precancerous lesions, which has certain clinical value. In this study, serum G-17 and PGI levels decreased significantly in patients after WMT treatment. There are no large-sample studies focusing on the effects of WMT on serum gastric function markers in healthy or gastric mucosal lesions, but based on its ability to remodel the microecology of many chronic intestinal diseases, WMT may serve as a potential adjunctive therapeutic strategy for gastric mucosal lesions, especially, *Helicobacter pylori*-associated diseases, and the changes in serum gastric function of the patients in the present study provide preliminary evidence supporting this hypothesis.

The observed changes in serum gastric function following WMT provide supportive evidence for this hypothesis. Although there is a lack of direct studies on the effect of WMT on serum gastric function in patients, several studies have indirectly shown the modulatory effect of WMT on serum gastric function. For example, a study on small intestinal bacterial overgrowth (SIBO) found that serum G17 levels were negatively correlated with hydrogen concentration in the small intestine, suggesting that the state of intestinal flora may influence gastric prohormone secretion through gastric acid secretion and neuroendocrine pathways (27). In terms of WMT for HP infection, studies have shown that WMT achieved an eradication rate of 40.6% in some patients who were not treated with anti-HP eradication therapy (28), suggesting that WMT contributes to the restoration of the mucosal barrier by inhibiting HP infection and reducing mucosal inflammation.

Meanwhile, the reduction in inflammatory markers following WMT suggests that systemic inflammatory activity was effectively attenuated. The observed positive correlation between changes in CRP and PGI further indicates a potential interaction between systemic inflammation and gastric mucosal status, implying that inflammatory regulation may influence gastric mucosal injury and repair processes. This association may be mediated through modulation of inflammatory signaling pathways such as NF-κB and IL-6, which are known to participate in gastric mucosal repair by regulating cell proliferation, apoptosis, and repair-related gene expression (28, 29). Accumulating evidence supports the anti-inflammatory effects of FMT. Both experimental and clinical studies have demonstrated that FMT can reduce pro-inflammatory cytokines including IL-6 and TNF-*α* while increasing anti-inflammatory mediators such as IL-10 (30, 31). In patients with ulcerative colitis, WMT has been shown to regulate TNF-α and IL-6 levels and rapidly decrease inflammatory indices (32). Similarly, reductions in CRP and PCT levels have been reported following FMT in antibiotic-associated diarrhea, Crohn’s disease, and related conditions, primarily through restoration of gut microbial balance and attenuation of systemic inflammatory responses (33–35). These findings provide mechanistic support for the inflammation-modulating role of microbiota-based interventions. It should be noted that decreases in serum PGI and G-17 levels do not exclusively indicate gastric mucosal repair, as similar alterations may occur under conditions of reduced gastric acid secretion or glandular atrophy. Therefore, changes in PGI and G-17 should be interpreted cautiously and in conjunction with other clinical and biochemical parameters. In the present study, when considered alongside concurrent reductions in inflammatory markers, improvements in gastrointestinal symptoms, and modulation of the gut microbiota, the observed biomarker changes are more likely to reflect attenuation of inflammatory activity rather than progressive glandular dysfunction. Nevertheless, given the observational design and the absence of endoscopic or histological confirmation, these findings should be regarded as suggestive rather than definitive. Further studies incorporating direct mucosal assessment are warranted. In addition to laboratory improvements, gastrointestinal symptom scores were significantly reduced after WMT treatment. Similar symptom benefits have been reported in other disease contexts. For example, WMT significantly improved gastrointestinal symptoms in patients with non-erosive reflux disease (NERD), reducing proton pump inhibitor dependence and prolonging remission (21). In children with autism, WMT alleviated constipation and diarrhea (36), and in patients with irritable bowel syndrome, abdominal pain and bloating scores decreased following treatment (37). Collectively, these findings suggest that microbiota-based modulation may contribute to symptomatic improvement, potentially through regulation of systemic and mucosal inflammatory responses.

This study demonstrated that WMT treatment significantly increased the *α*-diversity of the gut microbiota. The simultaneous increases in the Chao1, Simpson, and Shannon indices indicate improvements in both microbial richness and evenness, consistent with the proposed mechanism of WMT in restoring intestinal microbial homeostasis. Previous studies in patients with metabolic syndrome (38) and chronic disease–related anemia (39) have similarly reported that WMT not only enhances microbial diversity but also promotes the recovery of short-chain fatty acid (SCFA)–producing bacteria. At the compositional level, the increased abundance of Agathobacter and Erysipelotrichaceae_UCG-003 has been associated with activation of SCFA metabolic pathways, whereas the decreased abundance of Eggerthella and Weissella may reduce their pro-inflammatory effects (40, 41). Using Spearman correlation analysis, we further explored the dynamic associations between baseline serum gastric function parameters (G-17, PGI, and PGII), HP antibody levels, and fecal microbiota at the genus level. HP antibody levels were positively correlated with Escherichia–Shigella and Akkermansia, suggesting that HP infection may be accompanied by enrichment of pathogenic and mucin-degrading taxa. In addition, PGR was negatively correlated with Escherichia–Shigella, Klebsiella, Megasphaera, and Acidaminococcus, indicating that impaired gastric function may be associated with increased abundance of potential opportunistic pathogens. Notably, G-17 showed a significant positive correlation with Acidaminococcus, suggesting a potential microbiota–gastric endocrine interaction. Acidaminococcus, a member of the Veillonellaceae family, metabolizes amino acids and produces SCFAs, particularly acetate and propionate. SCFAs are increasingly recognized as key microbial signaling molecules capable of activating G-protein–coupled receptors on enteroendocrine cells, thereby modulating gastrointestinal hormone secretion, mucosal immune responses, and inflammatory pathways. Although direct causal evidence linking Acidaminococcus to gastrin secretion remains limited, SCFA-mediated signaling represents a biologically plausible mechanism underlying the observed association between microbial abundance and circulating G-17 levels. Given that gastrin release is tightly regulated by gastric acidity and mucosal feedback, alterations in microbial metabolites following WMT may indirectly influence the gastrin regulatory loop. Collectively, these findings provide microecological evidence supporting the association between gut microbiota alterations and gastric functional abnormalities and offer mechanistic insight into metabolic crosstalk along the “gut–stomach axis.”

**Figure 8:**
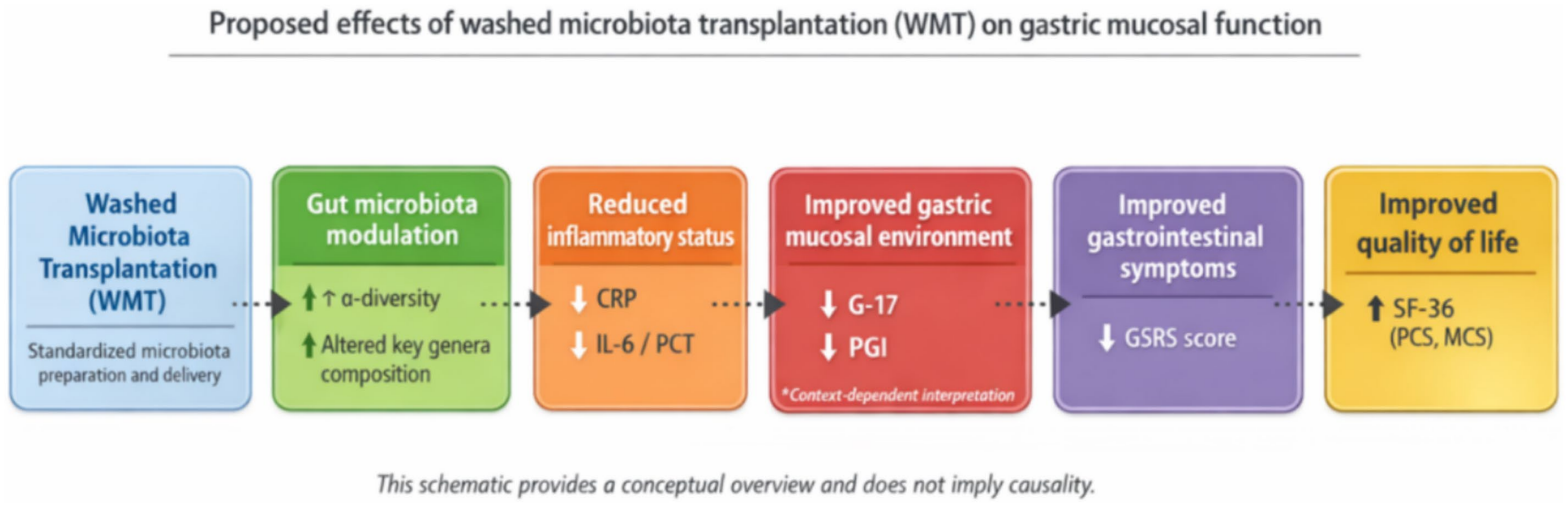
Conceptual schematic overview of the proposed effects of washed microbiota transplantation on gastric mucosal function.

In the assessment of patients ‘quality of life by WMT in this study, the improvement in SF-36 scores not only reflected the enhancement of physiological functioning, but also revealed positive changes in patients’ mental health. The positive correlation between symptom relief and improved quality of life (42), and symptom relief helps to reduce anxiety and depression induced by chronic discomfort, leading to an overall improvement in quality of life. For example, it has been noted that improvement in gastrointestinal symptoms reduces psychological stress and sleep disturbances, thereby enhancing quality of life (43). A study on WMT for inflammatory bowel disease pointed out that WMT not only improved sleep quality (through PSQI score reduction), but also alleviated clinical symptoms of IBD (e.g., abdominal pain, bloody stools, and diarrhea), thus indirectly enhancing quality of life. WMT affects sleep quality and psychological health through the gut-brain axis by improving the balance of intestinal flora, thus enhancing quality of life (44). Under strict donor screening, standardized preparation processes and medical supervision, FMT is considered safe and effective for indications such as recurrent *C. difficile* infection, and adverse events are dominated by mild symptoms (e.g., diarrhea, bloating) (45, 46). Some studies have shown that its adverse event rate for ulcerative colitis is not significantly different from that of the control group (47, 48). WMT further reduces the risk of diarrhea and infections by precisely controlling the number of flora and the washing process (49, 50). This study demonstrated that WMT has fewer adverse effects and good tolerability, and its mild therapeutic properties are more suitable for long-term use than traditional proton pump inhibitor or antibiotic therapy, suggesting potential clinical relevance. Comprehensive analysis suggests that WMT may reduce local and systemic inflammation levels by regulating the balance of intestinal flora, thereby promoting the repair of gastric mucosa and normalising G-17 and PGI indices. Although this hypothesis needs to be verified by further molecular mechanism studies, it provides a new perspective for our understanding of gastric mucosal injury and repair. In this study, we further explored the impact of WMT on serum gastric function markers: pepsinogen I, pepsinogen II, and gastrin-17, laying the foundation for subsequent research on the effects of environmental factors (51), gut microbiota (52–54), metabolic biomarkers (55, 56), and the influence of WMT (57–63) on diseases. A schematic summary of the proposed gut–stomach axis linking microbiota modulation, inflammation reduction, and gastric mucosal improvement is presented in Figure 8.

Given the relatively short follow-up period (approximately 5 months), the durability of the observed changes, including long-term maintenance, normalization, or recurrence, was considered at the study design stage. However, a comprehensive assessment of long-term outcomes was beyond the scope of the present study due to limitations in study duration and design. Accordingly, the results primarily reflect short-term changes following WMT. While the sample size was limited and long-term randomized comparisons were not available, the observed changes in serum gastric function parameters, inflammatory markers, and clinical symptoms during the follow-up period may suggest potential short-term effects of WMT. In addition, the comparison group was observational rather than a strictly matched or randomized control, and patient-reported outcome measures were not assessed in that group. As SF-36 and GSRS evaluations were performed only in the WMT cohort, changes in these subjective measures were based solely on within-group comparisons; therefore, potential expectancy or placebo-related effects cannot be excluded, and improvements in quality-of-life and symptom scores should be interpreted cautiously. Furthermore, the heterogeneity of underlying diseases and the lack of endoscopic or histological confirmation limit definitive conclusions regarding gastric mucosal structural changes.

These preliminary observations underscore the need for future large-scale, multicenter randomized controlled trials with extended follow-up to validate the durability and clinical significance of the observed Gastrin-17 reduction. Such studies should incorporate sham-controlled designs where feasible, standardized disease stratification, and comprehensive outcome measures—including endoscopic and histological evaluation, imaging, and expanded biochemical profiling—to clarify the long-term sustainability of gastric endocrine modulation and to better define the mechanisms and therapeutic potential of WMT in well-characterized patient populations.

## 5 Conclusion

This study demonstrates that Washed Microbiota Transplantation (WMT) is a safe and effective intervention for modulating gastric function markers and reducing systemic inflammation. While both WMT and routine treatments led to internal improvements in pepsinogen levels, WMT provided a statistically superior reduction in G-17 compared to the control (*p* = 0.032), suggesting a specific benefit for antral mucosal homeostasis. The observed shift in gut microbiota diversity, particularly the correlation between Acidaminococcus and G-17, suggests that the gut-gastric axis is a viable therapeutic target. These findings, combined with significant improvements in patient quality of life, support WMT as a promising complementary therapy for chronic gastric dysfunction, warranting further validation through large-scale randomized controlled trials.

## Data Availability

The original contributions presented in the study are publicly available. This data can be found in the NCBI SRA repository, under accession number: PRJNA1422256. All data generated or analysed during this study are available from the corresponding authors upon reasonable request.
